# Supplementary material for: The Effect of Diabetes on Prognosis Following Myocardial Infarction Treated with Primary Angioplasty and Potent Antiplatelet Therapy
Source: J Clin Med. 2020 Aug 6;9(8):2555. doi: 10.3390/jcm9082555 (PMC7464834; doi:10.3390/jcm9082555)
Supplement: Supplementary file 1 [file jcm-09-02555-s001.pdf]

Suppllement to:

THE EFFECT OF DIABETES ON PROGNOSIS FOLLOWING MYOCARDIAL INFARCTION TREATED WITH PRIMARY ANGIOPLASTY AND POTENT ANTIPLATELETS

Cardiovascular drugs and therapy journal

Stanislav Simek, MD, CSc; Zuzana Motovska, MD, PhD; Ota Hlinomaz, MD, CSc; Petr Kala, MD, PhD; Milan Hromadka, MD, PhD; Jiri Knot, MD, PhD; Ivo Varvarovsky, MD, PhD; Jaroslav Dusek, MD, PhD; Richard Rokyta, MD, PhD5; Frantisek Tousek, MD; Michal Svoboda, MSc; Alexandra Vodzinska MD; Jan Mrozek, MD; Jiri Jarkovsky, MSc, PhD; et al from PRAGUE-18 Study Group.

Corresponding Author: Zuzana Motovska, Cardiocenter, Third Faculty of Medicine, Chrales University and University Hospital Kralovske Vinohrady, Prague, Czech Republic; e-mail: motovska.zuzana@gmail.com, phone:+420 267163760, fax: +420 267163763 •

Figure S1: The occurence of stent thrombosis and serious bleeding in patients with and without diabetes mellitus (without diabetes mellitus, with diabetes mellitus on insulin therapy and not on insulin therapy) following acute myocardial infarction (AMI) treated with primary percutaneous coronary intervention and prasugrel or ticagrelor. (Time-to-event analysis was done using the Kaplan-Meier estimate of survival function.)

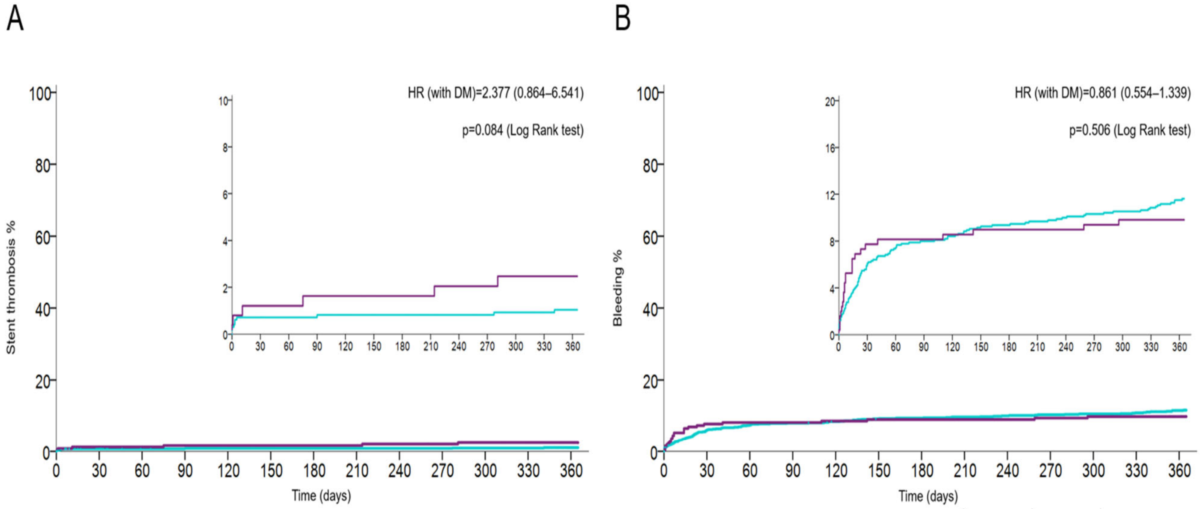

A

			30	180	365
no DM		No. at risk	953	936	824
		EP probability (95% CI)	0.7 (0.2; 1.2)	0.8 (0.3; 1.4)	1.0 (0.4; 1.7)
DM		No. at risk	238	233	204
		EP probability (95% CI)	1.2 (0.0; 2.6)	1.6 (0.0; 3.2)	2.5 (0.5; 4.4)

B

			30	180	365
no DM		No. at risk	903	856	743
		EP probability (95% CI)	6.1 (4.6; 7.6)	9.4 (7.5; 11.2)	11.6 (9.6; 13.6)
DM		No. at risk	223	217	190
		EP probability (95% CI)	7.7 (4.4; 11.1)	9.0 (5.4; 12.6)	9.8 (6.1; 13.6)

Supplementary list 1. List of Study Sites and Investigators

Study Principal Investigators: Zuzana Motovska and Petr Widimsky

1. Cardiocentre, Third Medical Faculty of Charles University and University Hospital Kralovske Vinohrady, Prague, Czech Republic:

Principal Investigator: Zuzana Motovska, MD., PhD.

Investigators: Jiri Knot MD. PhD., Jaroslav Ulman MD., Frantisek Bednar MD. PhD., Martin Kamenik MD., Petra Paulů MD. PhD., Dana Bilkova MD. PhD., Teodora Vichova MD. PhD., Robin Kralik MD., Karel Vondrak. MD, Vaclav Bufka MD., Assoc. Prof. Pavel Osmancik MD. PhD., Dalibor Herman MD. PhD., Petr Stros MD., Karol Curila MD. PhD., Assoc Prof. Petr Tousek MD. PhD., Tomas Budesinsky MD., Prof. Petr Widimsky MD. DrSc.

2. First Department of Internal Medicine - Cardioangiology, ICRC, Faculty of Medicine Masaryk University and St. Anne's University Hospital, Brno, Czech Republic

Principal investigator: Ota Hlinomaz MD, CSc

Investigators: Petra Kramariková Mgr., Marketa Beranová, Ladislav Groch MD. , Jan Sitar MD., Michal Rezek MD., Jiří Seménka MD., Martin Novák MD., Jiří Sikora MD., Blanka Fischerová MD.

3. Department of Internal Medicine and Cardiology, Faculty of Medicine Masaryk University and University Hospital Brno, Brno, Czech Republic.
Principal Investigator: Petr Kala MD. PhD. FESC.
Investigators: Roman Miklík MD. PhD., Lumir Koc MD., Petr Jerabek MD, Otakar Bocek MD., Roman Stipal MD. PhD., Jan Kanovsky MD PhD, Martin Poloczek MD, Robert Cyprian Mgr

4. Department of Cardiology, University Hospital and Faculty of Medicine in Pilsen, Charles University, Prague, Czech Republic.

Principal Investigator: Milan Hromadka MD. PhD.

Investigators: Prof. Richard Rokyta MD. PhD. FESC., Jan Pospisil MD.

5. Cardiology Centre AGEL, Pardubice, Czech Republic

Principal Investigator: Ivo Varvarovsky, MD. PhD.

Investigators: Martin Pavolko, MD., Martin Ráchela MD., Jan Málek MD., Vladimir Rozsíval MD.PhD., Vojtěch Novotný MD., Tomáš Lazarák MD., Jan Matějka MD.PhD.

6. First Department of Internal Medicine, University Hospital Hradec Kralove, Charles University in Prague, Faculty of Medicine in Hradec Kralove, Czech Republic

Principal Investigator: Jaroslav Dusek MD., PhD.

Investigators: Jan Hulka MD, Assoc. Prof. Josef Stasek MD. PhD.

7. Cardiocenter - Department of Cardiology, Regional Hospital, Ceske Budejovice, Czech Republic

Principal Investigator: Frantisek Tousek MD. FESC.

Investigators: Ladislav Pesl MD., Ales Kovarik, MD., Dita Novakova MD, Martina Zitova MD., Milan Slapnicka MD., Radek Krejcí MD., Tomas Romsauer MD., Tomas Sattran MD.

8. Cardiocenter, Regional Hospital, Karlovy Vary, Czech Republic

Principal Investigator: Bohumil Majtan

Investigators: Michal Padour MD., Alexandr Schee MD., Roman Ondrejcak MD., Zdenek Peroutka MD.

9. Second Department of Medicine - Department of Cardiovascular Medicine, First Faculty of

Medicine, Charles University in Prague and General University Hospital in Prague, Prague, Czech Republic.

Principal Investigator: Stanislav Simek MD. PhD.

Investigators: Assoc. Prof. Jan Belohlavek MD. PhD.

10. AGEL Research and Training Institue - Trinec Branch, Cardiovascular Centre, Podlesi Hospital, Trinec, Czech Republic

Principal Investigator: Marian Branny MD. PhD.

Investigators: Alexandra Vodzinska MD., Jindrich Cerny MD, Jan Indrak MD, Miroslav Hudec MD, Michal Palowski MD., Radim Spacek MD., Daniel Matous MD.

11. Cardiovascular Department, University Hospital Ostrava, Ostrava, Czech Republic

Principal Investigator: Jan Mrozek MD.

Investigators: Martin Porzer MD., Pavel Kukla MD.

12. Department of Cardiology, Krajska zdravotni a.s., Masaryk hospital and UJEP, Usti nad Labem, Czech Republic

Principal Investigator: Prof. Pavel Cervinka MD, PhD

Investigator: Andrej Kupec MD., Marian Bystron MD.

13. First internal cardiology clinic, University hospital Olomouc, Olomouc, Czech Republic

Principal Investigator: Jiri Ostransky MD.

Investigator: Martin Sluka MD.

14. Cardiocenter, Hospital na Homolce

Principal investigator: Assoc. Prof. Martin Mates MD CSc

Investigators: Bohumil Majtan MD, Pavel Formanek MD, Petr Kmonicek, Karel Kopriva MD., Ondrej Aschermann MD.
